# Immunophenotypic characterization of acute leukemia at a public oncology reference center in Maranhão, northeastern Brazil

**DOI:** 10.1590/S1516-31802011000600005

**Published:** 2011-12-01

**Authors:** Elda Pereira Noronha, Heliana Trindade Marinho, Erika Bárbara Abreu Fonseca Thomaz, Cintia Assunção Silva, Geni Lourdes Ramos Veras, Raimundo Antônio Gomes Oliveira

**Affiliations:** I MSc. Pharmacist in the Masters’ degree program on Mother and Child Health, Universidade Federal do Maranhão (UFMA), and Clinical Research Center, University Hospital, UFMA, São Luís, Maranhão, Brazil.; II PhD. Professor, Department of Public Health, Universidade Federal do Maranhão (UFMA), São Luís, Maranhão, Brazil.; III MD. Pediatric Oncologist, Instituto Maranhense de Oncologia Aldenora Bello (IMOAB), São Luís, Maranhão, Brazil.; IV MSc. Pediatric Oncologist, Instituto Maranhense de Oncologia Aldenora Bello (IMOAB), São Luís, Maranhão, Brazil.; V PhD. Professor, Department of Pharmacy, Universidade Federal do Maranhão (UFMA), and Pharmacist, Clinical Research Center, University Hospital, UFMA, São Luís, Maranhão, Brazil.

**Keywords:** Precursor cell lymphoblastic leukemia-lymphoma, Leukemia, myeloid, acute, Prevalence, Immunophenotyping, Prognosis, Leucemia-linfoma linfoblástico de células precursoras, Leucemia mielóide aguda, Prevalência, Imunofenotipagem, Prognóstico

## Abstract

**CONTEXT AND OBJECTIVES:**

The incidence of acute leukemia (AL) subtypes varies according to geographical distribution. The aim here was to determine the incidence of morphological and immunophenotypic AL subtypes in the state of Maranhão, Brazil, and to correlate the expression of aberrant phenotypes in children with acute lymphoblastic leukemia (ALL) with prognostic factors.

**DESIGN AND SETTING:**

Single prospective cohort study at a public oncology reference center in Maranhão.

**METHODS:**

Seventy AL cases were diagnosed between September 2008 and January 2010. For the diagnosis, complete blood cell counts, myelograms (at diagnosis and at the end of the induction phase), cytochemical analysis and immunophenotyping were performed.

**RESULTS:**

Among adult patients (n = 22), the incidence of AL types was: ALL (22.7%) and acute myeloid leukemia (AML) (77.3%). The subtype AML M0 occurred most frequently (29.4%). In children (n = 48), the types were: AML (18.7%), most frequently subtype AML M4 (33.4%); biphenotypic acute leukemia (BAL) (4.2%); and ALL (77.1%), including the subtypes B-ALL (72.9%) and T-ALL (27.1%). Among the children with ALL, there were no statistically significant differences between patients with and without aberrant phenotypes, in relation to hematological parameters and treatment response.

**CONCLUSION:**

This work demonstrates that the frequencies of AML M0 cases among adults and T-ALL cases among children in Maranhão were high. This suggests that there may be differences in AML subtype incidence, as seen with ALL subtypes, in different regions of Brazil. No association was found between the expression of aberrant phenotypes and prognostic factors, in children with ALL.

## RESUMO

**CONTEXTO E OBJETIVOS:**

A incidência dos subtipos de leucemias agudas (LA) tem mostrado variações em relação à distribuição geográfica. Objetivou-se determinar a incidência dos subtipos morfológicos e imunofenotípicos de LA no estado do Maranhão, Brasil, e relacionar a expressão de fenótipos aberrantes em crianças com leucemia linfoblástica aguda (LLA) com fatores prognósticos.

**TIPO DE ESTUDO E LOCAL:**

Estudo de coorte único prospectivo em um centro oncológico de referência público no Maranhão.

**MÉTODOS:**

Diagnosticaram-se 70 casos de LA no período de setembro de 2008 a janeiro de 2010. No diagnóstico, realizaram-se hemogramas, mielogramas (no diagnóstico e ao final da fase de indução), citoquímica e imunofenotipagem.

**RESULTADOS:**

Nos pacientes adultos (n = 22), as incidências dos tipos de LA foram: LLA (22,7%) e leucemia mieloide aguda (LMA) (77,3%), sendo o subtipo LMA M0 mais frequente (29,4%). Em crianças (n = 48): LMA (18,7%), subtipo LMA M4 mais frequente (33,4%), leucemia bifenotípica aguda (BAL) (4,2%) e LLA (77,1%), sendo os subtipos LLA-B (72,9%) e LLA-T (27,1%). Na LLA, em crianças, não se encontrou diferença estatisticamente significante entre pacientes com e sem fenótipos aberrantes, em relação aos parâmetros hematológicos e resposta ao tratamento.

**CONCLUSÃO:**

Esta pesquisa demonstra elevada frequência de casos de LMA M0 em adultos, bem como das LLA-T em crianças no Maranhão, sugerindo que podem haver diferenças na incidência dos subtipos das LMA, assim como dos subtipos de LLA, em diferentes regiões do Brasil. Não foi encontrada associação entre a expressão de fenótipos aberrantes e fatores prognósticos em crianças com LLA.

## INTRODUCTION

Acute leukemias comprise a heterogeneous group of diseases characterized by rapid and uncontrolled clonal expansion of progenitor cells of the hematopoietic system.^1^ They are the most common form of childhood neoplasia, and acute lymphoblastic leukemia (ALL) represents 75% of all such cases. This percentage is much lower in adults, in whom acute myeloid leukemias (AMLs) are more common. In children, the vast majority of ALL cases (80%–85%) are of precursor B-lineage and about 15% of all cases are of T-lineage.^2,3^ A small number of patients whose blasts simultaneously present antigens of the myeloid and lymphoid lineages are characterized as carriers of mixed, hybrid or biphenotypic acute leukemias (BALs).^4,5^

AML and ALL in which the blasts contain one or two antigens of another lineage, but do not meet the criteria for BAL, are known respectively as acute myeloid leukemias with anomalous lymphoid expression and acute lymphoblastic leukemias with anomalous myeloid expression.^6,7^ Occurrence of aberrant phenotypes (or anomalous expression) are reported to have variable frequency and their prognostic value is controversial.^8^

Early classification systems for acute leukemias were based only on cytomorphological and cytochemical investigations. Morphology still plays a central role, but current classification systems have incorporated immunophenotyping in order to achieve greater precision in delineating the hematopoietic lineage and differentiation stage of particular leukemias. Immunophenotyping is fundamental for classifying lymphoid malignancies and is also essential for recognizing several subtypes of acute myeloid leukemia (e.g. AML-M0 and AML-M7) and biphenotypic acute leukemias; for monitoring the responses to treatment, including detection of minimal residual disease (MRD); and for identifying markers with prognostic implication. The current World Health Organization (WHO) classification of tumors of hematopoietic and lymphoid tissues incorporates not only immunophenotyping but also cytogenetic and molecular characteristics that contribute towards defining biologically and clinically relevant leukemia subsets. However, it is neither necessary nor cost-effective to perform multiple studies on every specimen.^9-11^

Analysis of the incidence of leukemia subtypes across the world has revealed important variations in relation to geographical distribution, sex, age, ethnicity and socioeconomic status, thus suggesting that several etiological factors exist.^12,13^ Therefore, further studies on the frequencies of different subtypes of acute leukemias are of great importance, especially in regions with different socioeconomic characteristics such as the northeast of Brazil, where such data are scarce.

Unfortunately, before our group created an immunophenotyping service for the state of Maranhão, acute leukemias were not diagnosed through immunological markers, except in rare cases of patients whose relatives had the financial means to send their samples to a more advanced center. As a result, the vast majority of such patients were treated and given a prognosis on the basis of morphological and clinical findings alone, which may sometimes blur the choice of the most appropriate treatment.

## OBJECTIVES

The aim of the present study was to determine the incidence of different morphological and immunophenotypic subtypes of acute leukemia among patients referred to an oncology reference center in the state of Maranhão for treatment of acute leukemias, and to correlate the expression of aberrant phenotypes in children with acute lymphoblastic leukemia (ALL) with prognostic factors.

## METHODS

### Sample and setting

The present investigation was approved by the Research Ethics Committee of the University Hospital of Universidade Federal do Maranhão (UFMA) (number 115/2008). It included all consecutive patients who had been referred to the Oncology Reference Center during the study period, consisting of 73 adults (≥ 18 years) and children (< 18 years). The Oncology Reference Center is a public institution in São Luís, Maranhão, Brazil. They were diagnosed with *de novo* acute leukemia between September 2008 and January 2010. The morphological and immunophenotypic evaluations on the samples were performed at the Clinical Research Center of the University Hospital, Universidade Federal do Maranhão (UFMA). Patients who refused to take part or whose legal guardians did not allow their participation (n = 1) and cases of blast crisis chronic myeloid leukemia (n = 2) were excluded, thus resulting in 70 participants in this study. The following patient data were gathered: sex, age and occurrences of deaths of children with ALL before the end of the induction phase.

We evaluated the association between prognostic factors (white blood cell count, platelet count, hemoglobin level, peripheral blast percentage and response to treatment) and expression of aberrant phenotypes in children with ALL. This association could not be analyzed in cases of children (n = 9) and adults (n = 17) with AML, or among adults with ALL (n = 5), due to the small sample size.

### Diagnosis

The diagnosis of acute leukemia was made based on the complete blood cell counts, bone marrow aspirate smear, cytochemical tests (myeloperoxidase, alpha-naphthyl acetate esterase and periodic acid-Schiff) and immunophenotyping by means of flow cytometry. Bone marrow smears were performed by means of differential counting of 500 cells stained with May-Grünwald-Giemsa. The analysis was performed by two morphologists and a diagnosis of acute leukemia was made when the bone marrow blast count was greater than 20%. The myeloperoxidase cytochemical test was considered positive when 3% or more of the blasts showed positive staining granules. The periodic acid-Schiff (PAS) test was considered positive when the blasts showed granular block and partial or complete ring-type PAS staining. For alpha-naphthyl acetate esterase, positive findings in more than 20% of the blasts defined the types AML-M4 or M5. AML was classified in accordance with the French-American-British (FAB) criteria.^14-16^ The classifications of ALL and BAL were made based on the criteria of the European Group for the Immunological Characterization of Leukemias (EGIL).^6^ Only the morphological and immunophenotypic criteria for leukemia diagnosis were used in the present study, and cytogenetic data that are taken into considered by WHO were not used because this service is still unavailable in our state.

Samples of peripheral blood (n = 12) or bone marrow (n = 58) were collected in ethylenediaminetetraacetic acid (EDTA) for immunophenotyping. The samples were processed within four hours of collection.

For the immunophenotypic diagnosis of acute leukemias, a combination of two or three fluorochrome-conjugated monoclonal antibodies (MoAb) per tube was added to the samples. All the MoAbs were obtained from Becton Dickinson (San José, California, United States). They were conjugated with fluorescein isothiocyanate (FITC), phycoerythrin (PE) or peridinin chlorophyll protein (PerCP), and were directed to antigens for T cells (CD1a, CD2, cytoplasmic (c) CD3, CD4, CD5, CD7 and CD8), B cells (CD10, CD19, cCD22, cCD79a, superficial and cIgM), myeloid cells [CD13, CD33, CD117 and myeloperoxidase (MPO)], monocytes (CD14 and CD64), erythroid cells (alpha-glycophorin), platelet cells (CD61 and CD41a), non-specific lineage pan-leukocytes (CD45) and precursor cells [CD34, human leukocyte antigen-DR (HLA-DR) and terminal deoxynucleotidyl transferase (TdT)].

Membrane and intracytoplasmic labeling was performed using 1 x 10^6^ cells per tube. For membrane labeling, the samples were incubated with each antibody for 20 minutes. The erythrocytes were lysed with 2 ml of FACS lysing solution (Becton Dickinson, San José, California, United States), diluted to 1:10 and then washed with 2 ml of phosphate-buffered saline (PBS; pH = 7.4). For intracytoplasmic labeling, FACS permeabilizing solution (Becton Dickinson, San José) was used in accordance with the manufacturer's instructions.

Data acquisition and sample analysis was performed in a FACSCalibur flow cytometer (Becton Dickinson, San José), using the CellQuest software (Becton Dickinson, San José), after calibration with the Calibrite bead kit (Becton Dickinson, San José), using the FACSComp program (Becton Dickinson, San José).

**Figure 1.**
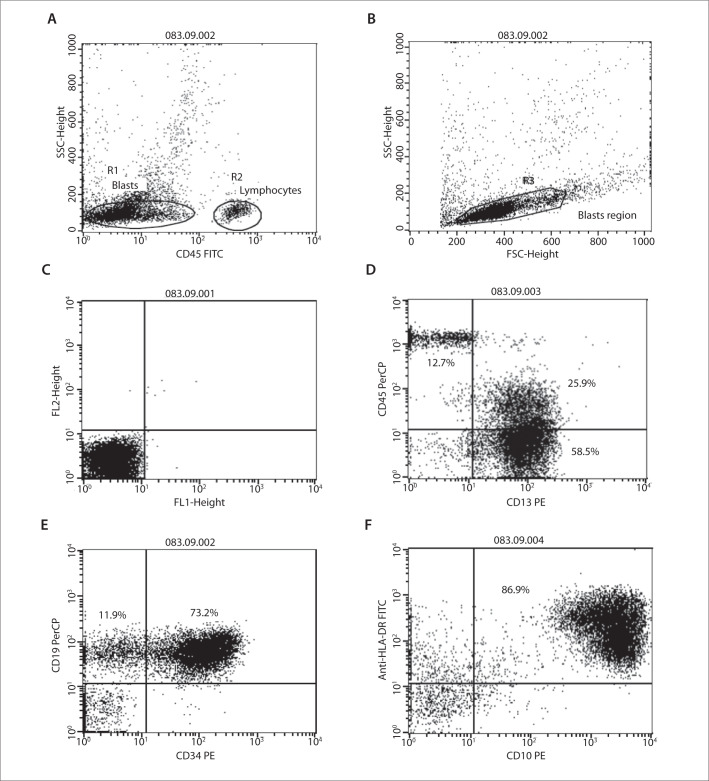
Immunophenotyping of B-ALL with aberrant antigen expression of CD13 in a patient at the Oncology Reference Center, São Luís, Maranhão. a) Dot plot of CD45 expression versus intracellular complexity (side scatter angle, SSC), differentiating the blast region (R1), with partial loss of CD45 expression and lymphocyte region (R2); b) Blast region (R3) in dot plot considering cell size (forward scatter angle, FSC) versus SSC; c) Analysis on negative controls (isotype IgG1 and IgG2a monoclonal antibodies); d) Positivity for the myeloid marker CD13 (25.9% + 58.5% = 84.4%) and for CD45 (25.9%); e) Positivity for the immature cell marker CD34 (73.2%) and for the B cell marker CD19 (73.2%); f) Positivity for the immature cell marker HLA-DR (86.9%) and for the B cell marker CD10 (86.9%).

Lymphocyte labeling with CD4 FITC/CD8 PE/CD3 PerCP was used, to compensate for fluorescence and eliminate overlapping before data acquisition. The blast gating strategy included using dot plots of CD45 expression versus intracellular complexity (side scatter angle, SSC) (Figure 1a) and also a second gate considering cell size (forward scatter angle, FSC) versus SSC (Figure 1b). A total of 10,000 events were acquired in the target gate. Negative controls using isotype IgG1 and IgG2a monoclonal antibodies were run in all cases. The criteria used for determining antigen positivity included analysis of negative controls (Figure 1c) and expression of the marker by more than 20% of the gated cells (Figure 1d, 1e and 1f). Similarly, aberrant phenotypes (Figure 1d) were defined when at least 20% of the blast cells expressed the particular aberrant marker. In ALL, aberrant expression of CD33 and CD13 was analyzed while in AML, aberrant expression of CD2, CD7 and CD19 was analyzed.

### Chemotherapy for children with ALL

Patients with ALL aged under 18 were treated in accordance with the protocol of the Brazilian Group for the Treatment of Childhood Leukemias (Grupo Brasileiro de Tratamento das Leucemias Infantis, GBTLI/99^17^). Patients with B-ALL were treated during the induction phase with dexamethasone (6 mg/m^2^/day), three doses per day for four weeks; vincristine (1.5 mg/m^2^/week) and daunorubicin (25 mg/m^2^/week) on days 0, 7, 14 and 21; L-asparaginase (5000 IU/m^2^/day), nine doses beginning between days 3 and 5 and administered three times per week; and MADIT (combination of methotrexate, cytarabine and dexamethasone administered intrathecally) with doses adjusted according to the patient's age and administered on days 0, 14 and 28.^17^ Patients with T-ALL were treated with the same drugs described above, although with the following modifications: daunorubicin (35 mg/m^2^/dose) on days 0, 28 and 42, and methotrexate (1 g/m^2^/dose) on days 7 and 21.

### Remission criteria among children with ALL

The response to treatment among children with ALL was evaluated by means of a bone marrow aspirate smear at the end of the induction phase and the presence of less than 5% blasts in the bone marrow was considered to be a criterion for remission. Patients who died before the end of the induction (n = 10) were considered to be patients who did not go into remission.

### Statistical analysis

An initial exploratory and descriptive analysis of the data was performed. The Shapiro-Wilk test was used to confirm the normal distribution of the quantitative variables. The variables studied presented asymmetrical distribution, and consequently, nonparametric tests were used. The hematological parameters of patients with and without aberrant phenotypes were compared using the Mann-Whitney test. The response to induction and the frequency of aberrant phenotypes compared between B-ALL and T-ALL, as well as between ALL and AML, were assessed by applying Fisher's exact test and the chi-square test. The significance level was taken to be 5% (P < 0.05) in all tests.

## RESULTS

A total of 70 patients with *de novo* acute leukemia were studied. Table 1 shows the incidence of the types of leukemias in the population studied. ALL was more frequent in children (77.1%) and AML was more common in adults (77.3%). BAL accounted for 2.9% of the cases of acute leukemias.

**Table 1.** Frequency of each type of acute leukemia, according to age group, among patients at the Oncology Reference Center, São Luís, Maranhão

Type of acute leukemia	Total cases n = 70	Children n = 48 (68.6%)	Adults n = 22 (31.4%)
Acute lymphoblastic leukemia	42 (60%)	37 (77.1%)	5 (22.8%)
Acute myeloid leukemia	26 (37.1%)	9 (18.7%)	17 (77.3%)
Biphenotypic acute leukemia	2 (2.9%)	2 (4.2%)	-

B-ALL was found in 72.9% (27/37) of the cases of ALL in children, while T-ALL accounted for 27.1% (10/37) of the cases. The immunological subtype ALL-BII (common B) was the most frequent type (51.6%; 16/31) among the cases of B-ALL (Table 2).

**Table 2.** Distribution of acute lymphoblastic leukemia (ALL) subtypes and patients’ sex at the Oncology Reference Center, São Luís, Maranhão

Immunological subtypes of ALL	Sex M/F	Total cases	Children	Adults
B-ALL	23/8	31 (73.8%)	27 (72.9%)	4 (80%)
ALL-BI*	3/0	3 (9.7%)	3 (11.1%)	-
ALL-BII*	10/6	16 (51.6%)	14 (51.8%)	2 (50%)
ALL-BIII*	7/2	9 (29%)	8 (29.6%)	1 (25%)
ALL-BIV*	2/0	2 (6.5%)	2 (7.4%)	-
B-ALL†	1/0	1 (3.2%)	-	1 (25%)
T-ALL	8/3	11 (26.2%)	10 (27.1%)	1 (20%)
**ALL Total**	**31/11**	**42 (100%)**	**37 (100%)**	**5 (100%)**

M = male; F = female;

* The value refers to the percentage of each subtype, calculated from the total number of cases of ALL-B in each age group;

† One case could not be subclassified in terms of the phase of maturation due to scarcity of material for evaluation.

The median age of the children with B-ALL was four years (range from one to 16 years), while that of children with T-ALL was eight years (range from two to 17 years). The peak incidence of ALL in children occurred between the ages of one and four years, representing 40.5% (22/37) of the cases of ALL diagnosed in children. Among the adults, the median age of those with ALL was 24 years (range from 19 to 56 years). Among the patients under the age of 18 years with ALL, the male-to-female ratio was 3.1:1.0. For B-ALL and T-ALL considered separately, the ratios were 3.5:1.0 and 2.3:1.0, respectively.

The hematological characteristics of the subtypes of ALL are presented in Table 3. It can be seen that T-ALL presented higher white cell counts, with a median greater than 50 x 10^9^/l. Regarding the hemoglobin level, ALL-BI (pro-B) presented a lower median (6.4 g/dl). All of the subtypes exhibited thrombocytopenia, with a median of less than 50 x 10^9^ platelets/l.

**Table 3.** Hematological parameters of the complete blood cell counts of acute lymphoblastic leukemia (ALL) subtypes on diagnosis, among patients at the Oncology Reference Center, São Luís, Maranhão

Hematological parameters	ALL subtypes				
ALL-BI	ALL-BII	ALL-BIII	ALL-BIV	T-ALL
Leukocytes x 10^9^/l	23.2 (2.35-102)	19.5 (1.44-157)	3.1 (1.13-135)	4.6 (2.3-6.9)	81.0 (9.1-591)
Hemoglobin g/dl	6.4 (6.0-6.6)	8.3 (3.4-12.9)	8.8 (7.0-10.9)	11 (9.3-12.7)	9.4 (6.5-12.5)
Platelets x 10^9^/l	12.0 (12-13)	18.0 (6.0-111)	45.0 (22-160)	33.5 (29-38.1)	39.0 (14-157)
% blasts	70.0 (46-96)	65 (0-97)	21.0 (0-99)	5.0 (4-6)	88.0 (4-98)

Median values (range).

Among the markers used to characterize B-ALL in cytoplasm, CD79a and CD22 presented the highest positivity rate (100%), followed by CD19 in the cell membrane (86.6%). CD10 and the immature cell marker HLA-DR presented positivity rates of 83.3% and 95%, respectively. The marker IgM in cytoplasm (cyt IgM) had a positivity rate of 33% and was positive in ten cases, which were classified as BIII-ALL (pre-B). CD34, an immature cell marker, was expressed in 61.2% of the cases and CD45 in 87%. Of the myeloid markers expressed anomalously, CD13 was the most frequent (48.2%).

In the immunophenotypic characterization of T-ALL, the markers CD3 (cytoplasm), CD5, CD7 and CD8 exhibited positivity rates of 100%. The markers CD2, CD1a, CD4, HLA-DR and CD10 showed positivity rates of 85.7%, 57.1%, 40%, 10% and 9.1%, respectively. CD34 was expressed in 54.5% of the cases and CD45 (with moderate to weak expression) was present in all cases. CD13 was the most frequent anomalous myeloid marker (36.3%).

There were aberrant phenotypes in 48.4% of the cases of B-ALL (15/31) and 36.4% (4/11) of the cases of T-ALL. There was no statistically significant difference between B-ALL and T-ALL in relation to the frequency of aberrant phenotypes (Table 4).

**Table 4.** Frequency of aberrant phenotypes in B-ALL and T-ALL patients at the Oncology Reference Center, São Luís, Maranhão

Subtype of ALL	Aberrant phenotypes	Total
Absent	Present
B-ALL	16 (51.6%)	15 (48.4%)	31
T-ALL	7 (63.6%)	4 (36.4%)	11
**Total**	**23**	**19**	**42**

ALL = acute lymphoblastic leukemia; Fisher's exact test was applied; P = 0.726.

No statistically significant difference was found in relation to prognostic factors (white blood cell count, platelet count, hemoglobin level, peripheral blast percentage and response to induction phase) in ALL in children with and without aberrant phenotypes (Table 5), although the number of patients who went into remission was higher in the group with aberrant phenotypes (ALL My^+^) (80%), compared with the group without aberrant phenotypes (ALL My^-^) (62%).

**Table 5.** Assessment of aberrant phenotypes in acute lymphoblastic leukemia (ALL), in relation to hematological parameters and response to induction, among children treated at the Oncology Reference Center, São Luís, Maranhão

Hematological and clinical parameters	P	ALL My^+^ n = 15	ALL My^-^ n = 22
Leukocytes x 10^9^/L*	0.361	41.2 (1.13-591.0)	13.65 (1.44-172.0)
Hemoglobin x g/dl*	0.757	9.1 (5.6 -11.0)	8.35 (5.1-12.9)
Platelets x 10^9^/l*	0.938	22.0 (6.0-160.0)	32.5 (8.0-137.0)
% blasts in peripheral blood*	0.268	70.0 (2-99)	60.0 (0-96)
Patients in remission/patients not in remission†	0.295	12/3	13/8‡

ALL My^+^ = acute lymphoblastic leukemia with aberrant phenotypes; ALL My^-^ = acute lymphoid leukemia without aberrant phenotypes; hematological parameters = values expressed as medians (range);

* Mann-Whitney test applied;

† Fisher's exact test applied;

‡ one patient abandoned the treatment before the end of induction and was excluded from this analysis.

The distribution of AML according to the FAB criteria revealed that the AML M4 subtype (3/9; 33.4%) was the one most frequently occurring in children. In the adults, the predominant subtype was AML-M0 (5/17; 29.4%) (Table 6).

**Table 6.** Frequency of French-American-British (FAB) subtypes and patients’ sex among acute myeloid leukemia (AML) cases at the Oncology Reference Center, São Luís, Maranhão

FAB subtypes of AML	Sex M/F	Total cases	Children	Adults
AML-M0	5/1	6 (23.1%)	1 (11.1%)	5 (29.4%)
AML-M1	0/4	4 (15.4%)	2 (22.2%)	2 (11.8%)
AML-M2	3/2	5 (19.2%)	1 (11.1%)	4 (23.5%)
AML-M3	1/4	5 (19.2%)	1 (11.1%)	4 (23.5%)
AML-M4	5/0	5 (19.2%)	3 (33.4%)	2 (11.8%)
AML-M6	1/0	1 (3.9%)	1 (11.1%)	-
**Total**	**15/11**	**26 (100%)**	**9 (100%)**	**17 (100%)**

M = male; F = female.

The adult patients presented a median age of 35 years (range: 19-67 years), while the median age among the children was 8 years (range: 1-15 years). The ratio of males to females with AML was 1.3:1.

Table 7 shows the hematological characteristics of the AML subtypes. It can be seen that AML-M1 presented a higher white blood cell count with a median of 50.5 x 10^9^/l. In relation to the hemoglobin level, the subtypes of leukemias presented medians with very similar values, varying between 7.1 g/dl (AML- M0) and 8.5 g/dl (AML-M1). All of the subtypes exhibited thrombocytopenia.

**Table 7.** Hematological parameters of the complete blood cell counts for acute myeloid leukemia subtypes among patients at the Oncology Reference Center, São Luís, Maranhão

Hematological parameters	Acute myeloid leukemia subtypes
M0	M1	M2	M3	M4
Leukocytes x 10^9^/l	6.4 (1.2-189.0)	50.5 (3.1-266.0)	3.2 (2.2- 8.3)	14.4 (2.1-55.9)	24.2 (2.1-100.0)
Hemoglobin /dl	7.1 (5.8-15.1)	8.5 (8.0-9.2)	7.8 (4.3-11.1)	7.3 (1.6-10.0)	7.8 (3.5-11.6)
Platelets x 10^9^/l	63.0 (8.0-129.0)	40.0 (4.0-253.0)	29.0 (5.0-77.6)	12.0 (10.0-26.0)	62.0 (12.0-257.0)
% blasts	82.5 (60.0-93.0)	68.5 (23.0-97.0)	16.5 (13.0-20.0)	31.0 (2.0-98.0)	14.0 (5.0 -73.0)

Values expressed as medians (range).

Among the markers used to characterize AML, CD117 and CD13 exhibited the highest positivity rates (100%), followed by CD33 (96.1%). MPO presented a positivity rate of 73.9%, while CD14 and CD64 presented positivity rates of 29.1% and 33.3%, respectively. The immature cell markers CD34, HLA-DR and CD117 had positivity rates of 69.2%, 63% and 100% respectively. Out of the five cases diagnosed with AML-M3, only one expressed CD34, and none expressed HLA-DR. CD45 was expressed in 92.3% of the cases.

There were aberrant phenotypes in 27% of the diagnosed cases, and expression of CD7 occurred most frequently (19.2%). There was no statistically significant difference between ALL and AML in relation to the frequency of aberrant phenotypes (Table 8).

**Table 8.** Frequency of aberrant phenotypes in acute lymphoblastic leukemia and acute myeloid leukemia among patients at the Oncology Reference Center, São Luís, Maranhão

ALL subtype	Aberrant phenotypes		Total
	Absent	Present	
Acute lymphoblastic leukemia	23 (54.7%)	19 (45.3%)	42
Acute myeloid leukemia	19 (73%)	7 (27%)	26
Total	42	26	68

The chi-square test was applied; P = 0.199.

## DISCUSSION

This work constitutes the first study to be carried out in the state of Maranhão involving characterization of the immunophenotypic profile of cases of acute leukemias, and thereby determining the various immunological subtypes of these pathological conditions. The majority of ALL cases (88.1%) examined were diagnosed in children, and this matches the epidemiological data already described, which showed much lower frequencies of this neoplasia in adults.^18,19^ In the present investigation, the cases of ALL in adults represented just 22.7% of all the cases of acute leukemias in this age group.

The incidence of ALL is higher among men than among women, independent of the age group analyzed. In a study involving children from the states of Rio de Janeiro and Bahia and the Federal District, the ratio of males to females was 1.2:1.^13^ In the state of Pernambuco, this ratio was 1.7:1,^20^ while in Ribeirão Preto (city in the state of São Paulo), it was 1.8:1 for all subtypes of ALL, with an even higher predominance of males for T-ALL, at 4.2:1.^21^ In our work, for all subtypes of ALL, we found a higher proportion of males (3.1:1) than what has been described in other Brazilian state of, with a higher male-to-female ratio for B-ALL (3.5:1) than for T-ALL (2.3:1).

In relation to the subtypes of ALL, the present study revealed that 73.8% of the cases were classified as B-ALL and 26.2% of the cases as T-ALL. Among the B-ALL subtypes, ALL-BII (common B) occurred most frequently. These results are similar to those of other studies, except for the high frequency of T-ALL in children. In the population of Maranhão, the children presented a higher frequency of T-ALL (27%) than what is generally described in the literature (7.3% to 16%).^13,21,22^ Nevertheless, in another study involving children, Bachir et al.^23^ observed a frequency of 21.1% for T-ALL in Morocco, while in a study carried out in India by Rajalekshmy et al.,^24^ it was found that the frequency of T-ALL in children was 45.9%. In Recife (Brazilian state of Pernambuco), this frequency was 18.5%,^20^ similar to what was found in the state of Minas Gerais, Brazil (18%).^25^

A high frequency of T-ALL is associated with poor socioeconomic conditions, as demonstrated by research conducted in Brazil, which found a direct association between poor socioeconomic status (low *per capita* income) and the T phenotype.^25^ Another factor associated with increased frequency of T-ALL is ethnic origin, since the T phenotype has been shown to be more frequent among nonwhites.^13^ Taking into consideration that the state of Maranhão is the second poorest in Brazil,^26^ and that the population is predominantly nonwhite,^27^ this may explain the high incidence of T-ALL found in the present investigation.

In relation to AML, our results show that this disease was much more frequent among adults (65.4%) than in children (34.6%), and this finding is in agreement with reports in the literature.^28^

With regard to another aspect of our findings, taking into consideration all of the patients, our results indicate a much lower frequency of AML (37.1%) than of ALL (60%). A similar result was described by Rego et al.,^18^ who found that in the state of Piauí, the frequency of AML was half that of ALL.

In relation to the age of the patients with AML, the median obtained for the population of Maranhão was lower than that of patients with AML in developed countries,^29,30^ but was similar to that observed in other studies conducted in Brazil.^18,31,32^ This can be explained by the fact that, in developed countries, life expectancy is higher and elderly individuals constitute a greater proportion of the population. This differs from Brazil, especially in the northeast of the country, where life expectancy is low and, consequently, the elderly population is much smaller, as reported by Rego et al.^18^ Since the highest incidence of AML is seen in people over 60 years of age,^28^ this may explain the low incidence of the disease in the population of Maranhão.

Analysis on the distribution of the FAB subtypes revealed predominance of AML- M4 followed by subtype AML-M1 in children. This result is similar to that found in research performed in São Paulo,^33^ while a higher frequency of subtypes M2 and M3 was reported in Minas Gerais.^34^ In contrast, work by the Berlin-Frankfurt-Münster (BFM) group in Germany showed higher frequency of subtypes M4 and M5.^35^

Among the adults, the most frequent subtype was M0, followed by subtypes M2 and M3, which presented equal frequency. Aside from the M0 subtype, which is considered to be a less common form of leukemia comprising approximately 2% to 3% of myeloid leukemias,^36^ the M2 and M3 subtypes are very frequent in the Brazilian population.^18,31,32,37^ According to Rego et al.,^18^ the distribution of FAB subtypes is irregular, showing large geographical variations possibly as a result of ethnic and environmental factors.

Other studies carried out in Brazil have indicated differences with regard to the distribution of morphological subtypes of acute leukemias. In comparison with rates in Campinas (state of São Paulo) and Teresina (state of Piauí), it was found that the most common subtype in Teresina was M2, followed by M4 and M5 with equal frequencies, while in Campinas there was higher frequency of the M4 subtype followed by the M3 subtype.^18^ In São José dos Campos (state of São Paulo), there was higher prevalence of the M1 subtype, followed by M2.^37^ In Rio de Janeiro, there was higher frequency of M2, followed by M3 and M4, which were equally frequent,^31^ while in Rio Grande do Sul, higher frequency of M2 was found, followed by M1.^32^

In the present study, the increased incidence of the FAB subtype M0 was based on samples obtained from a single institution. It should be emphasized that the patients studied may not be representative of the whole region. Similar problems have affected other studies.^31,32,37^ In addition, the majority of the studies^18,31,32^ that have described the distribution of FAB morphological subtypes were retrospective, carried out by analyzing the medical records, many of which do not include immunophenotyping as a diagnostic technique for AML. In other cases, the immunophenotypic diagnosis was introduced a long time after the beginning of data collection, which probably means that the frequency of FAB M0 subtypes in the Brazilian population has been underestimated.

Numerous studies in developed countries, which used not only morphology and cytochemistry but also immunophenotyping, found relatively higher frequencies of the M0 subtypes among AML cases, as reported for example by Suárez et al.^38^ in Europe, Kaleem et al.^39^ in the United States and Chang et al.^29^ in Canada, who found frequencies of 15%, 17.6% and 9.5%, respectively.

The frequency of abnormal expression of an antigen from a given lineage in another lineage (aberrant phenotypes) is very variable in acute leukemias.^8^ In our study, 45.2% of the cases of ALL exhibited aberrant phenotypes, and the most frequent marker was CD13. This was similar to the results of Putti et al.,^40^ Den Boer et al.^41^ and Bachir et al.^23^ The frequency of myeloid coexpression was higher in B-ALL cases (48.4%) than in T-ALL cases (36.4%), although the difference was not significant. Den Boer et al.^41^ and Abdelhaleem^42^ also found a higher frequency in B-ALL cases, but only the latter found the difference to be significant. In AML cases, there was anomalous expression in 26.9% of them, and the most frequent marker was CD7, thus confirming the findings of Zheng et al.^43^

The association between prognostic factors and aberrant phenotypes in ALL in children remains controversial. Putti et al.^40^ and Pui et al.^44^ did not find any significant association with adverse prognostic factors. Riley et al.^45^ stated that aberrant phenotypes in ALL, both in adults and in children, were significantly associated with short duration of event-free survival, short duration of first remission and high relapse rates within the treatment phase.

In our analysis of cases of ALL in individuals under the age of 18 years, there was no difference between patients with and without aberrant phenotypes in relation to prognostic factors, although the number of patients who achieved remission was greater in the group with anomalous expression. This is similar to the results found by Bhushan et al.^8^

The literature shows great variation of data in relation to the frequency of anomalous expression in acute leukemia cases, and this may explain the lack of consistency with regard to the prognostic value of aberrant phenotypes in acute leukemia. Such variation may have multiple causes, including the use of different fluorochromes, the use of different clones of monoclonal antibodies, the sample characteristics, the technique and form of analysis used, the numbers and characteristics of patients, and the treatment protocol adopted.

## CONCLUSION

The use of immunophenotyping in our study made it possible to diagnose cases of minimally differentiated acute myeloid leukemia (AML-M0) and to differentiate B-ALL from T-ALL. The study showed that T-ALL and AML-M0 occurred at higher frequency in the population studied, thus suggesting that there may be differences in the incidence of the FAB subtypes of AML, as well as in the subtypes of ALL, in different regions of Brazil. Furthermore, in all of these cases, the lack of immunophenotypic analysis could have compromised the diagnosis and, as a result, compromised the choice of the most appropriate treatment. We did not find any association between aberrant phenotypes and the prognostic factors and clinical outcomes. The evaluations on the parameters examined in this work, such as aberrant phenotypes, leukemia subtypes and patient survival, can be improved; however, this requires longer duration of observation, and this should be borne in mind in future investigations.
